# Cohort Profile: ZOE 2.0—A Community-Based Genetic Epidemiologic Study of Early Childhood Oral Health

**DOI:** 10.3390/ijerph17218056

**Published:** 2020-11-01

**Authors:** Kimon Divaris, Gary D. Slade, Andrea G. Ferreira Zandona, John S. Preisser, Jeannie Ginnis, Miguel A. Simancas-Pallares, Cary S. Agler, Poojan Shrestha, Deepti S. Karhade, Apoena de Aguiar Ribeiro, Hunyong Cho, Yu Gu, Beau D. Meyer, Ashwini R. Joshi, M. Andrea Azcarate-Peril, Patricia V. Basta, Di Wu, Kari E. North

**Affiliations:** 1 Division of Pediatric and Public Health, Adams School of Dentistry, University of North Carolina-Chapel Hill, NC 27599-7450, USA; Gary_Slade@unc.edu (G.D.S.); Jeannie_Ginnis@unc.edu (J.G.); simancas@email.unc.edu (M.A.S.-P.); cagler@email.unc.edu (C.S.A.); poojansh@live.unc.edu (P.S.); deepti_karhade@unc.edu (D.S.K.); 2 Department of Epidemiology, Gillings School of Global Public Health, University of North Carolina-Chapel Hill, NC 27599-7400, USA; patricia_basta@unc.edu (P.V.B.); kari_north@unc.edu (K.E.N.); 3 Department of Comprehensive Dentistry, School of Dental Medicine, Tufts University, Boston, MA 02111, USA; Andrea.Zandona@tufts.edu; 4 Department of Biostatistics, Gillings School of Global Public Health, University of North Carolina-Chapel Hill, NC 27599-7400, USA; jpreisse@bios.unc.edu (J.S.P.); hunycho@live.unc.edu (H.C.); yugu@live.unc.edu (Y.G.); did@email.unc.edu (D.W.); 5 Division of Diagnostic Sciences, Adams School of Dentistry, University of North Carolina-Chapel Hill, NC 27599-7450, USA; apoena@email.unc.edu; 6 Division of Pediatric Dentistry, College of Dentistry, The Ohio State University, Columbus, OH 43210, USA; meyer.781@osu.edu; 7 Division of Surgery, School of Medicine, University of North Carolina-Chapel Hill, NC 27599-7050, USA; ashwini1@email.unc.edu; 8 Center for Gastrointestinal Biology and Disease, Division of Gastroenterology and Hepatology, and UNC Microbiome Core, Department of Medicine, School of Medicine, University of North Carolina-Chapel Hill, NC 27599-7555, USA; andrea_azcarate-peril@med.unc.edu; 9 Division of Oral and Craniofacial Health Sciences, Adams School of Dentistry, University of North Carolina-Chapel Hill, NC 27599-7450, USA; 10 Carolina Center for Genome Sciences, University of North Carolina-Chapel Hill, NC 27514, USA

**Keywords:** children, early childhood caries, community-based studies, oral health, genomics

## Abstract

Early childhood caries (ECC) is an aggressive form of dental caries occurring in the first five years of life. Despite its prevalence and consequences, little progress has been made in its prevention and even less is known about individuals’ susceptibility or genomic risk factors. The genome-wide association study (GWAS) of ECC (“ZOE 2.0”) is a community-based, multi-ethnic, cross-sectional, genetic epidemiologic study seeking to address this knowledge gap. This paper describes the study’s design, the cohort’s demographic profile, data domains, and key oral health outcomes. Between 2016 and 2019, the study enrolled 8059 3–5-year-old children attending public preschools in North Carolina, United States. Participants resided in 86 of the state’s 100 counties and racial/ethnic minorities predominated—for example, 48% (*n* = 3872) were African American, 22% white, and 20% (*n* = 1611) were Hispanic/Latino. Seventy-nine percent (*n* = 6404) of participants underwent clinical dental examinations yielding ECC outcome measures—ECC (defined at the established caries lesion threshold) prevalence was 54% and the mean number of decayed, missing, filled surfaces due to caries was eight. Nearly all (98%) examined children provided sufficient DNA from saliva for genotyping. The cohort’s community-based nature and rich data offer excellent opportunities for addressing important clinical, epidemiologic, and biological questions in early childhood.

## 1. Introduction

Dental caries is the most common chronic childhood disease worldwide. Early childhood caries (ECC) is an early-onset, aggressive form of the disease that affects an estimated 600 million children worldwide. Management of ECC is challenging—it frequently requires restorative care under sedation or general anesthesia, and there are populations wherein it remains largely untreated [1]. The disease imposes substantial human and economic costs on children, their families, and the public health care infrastructure [2]. This burden falls disproportionately on poor families, whose children are more likely to develop ECC compared to their non-poor counterparts, and who are frequently unable to access the requisite dental care [3,4].

ECC, similar to other forms of dental caries, is characterized by a biofilm-mediated, sugar-driven, multifactorial, dysbiotic shift at the interface of a tooth surface and its biofilm, which results in the progressive demineralization of dental hard tissues [5,6]. Despite a general decrease in caries prevalence in permanent teeth of older children and adults, the proportion of preschool children with ECC has persisted in recent decades [7]. This suggests that public health- and personal-preventive care, while effective in preventing caries in older children, has had little impact on ECC. An explanation for its persistence therefore must look beyond established behavioral, environmental, and societal risk factors. A better understanding of genetic factors underlying individual susceptibility and its interaction with oral health-related behaviors and the environment is needed [8].

Earlier observations emanating from twin, family, candidate gene, and recent genome-wide association studies (GWAS) support a considerable genetic component for dental caries [8,9,10]. Our group recently estimated ECC heritability to be 44% but this estimate was based on a very small sample size and was imprecise [11]. More recently, Haworth and colleagues [12] reported heritability estimates for primary dentition caries based on a consortium (*n* = 7230) meta-analysis to be 28% (95% confidence interval: 9–48%). Despite this evidence, efforts to identify genetic susceptibility factors associated with ECC have not paralleled recent genomics discoveries for dental caries among adults [13]. The first GWAS of ECC involved primarily European–American (white) children [14], whereas the recent consortium-based analysis [12] included small numbers of non-European children. Data collection and the resulting phenotypic heterogeneity was a limitation of the consortium GWAS, and both studies included predominantly children over the age of six, whereas 71 months is the uppermost age threshold for defining ECC [1]. Despite these issues, both studies provided evidence in support of loci implicated in childhood caries including *ALLC*, *MPPED2*, and *PKD2*. 

The genome-wide association study of ECC (“ZOE 2.0”) is a community-based, multi-ethnic, genetic epidemiologic study of early childhood oral health seeking to add to the knowledge base of ECC genomics. The justification of ZOE 2.0 was supported by four key features: (1) a focus on 3–5-year-old children, an age range within which the ECC phenotype is defined and clinically-determined caries experience has been the least distorted by restorative treatment and shedding of primary teeth; (2) a large sample of >6000 children; (3) a community-based (non-clinical) sample; (4) a tested clinical examination protocol including tooth-surface level recording of lesions of different severity levels. Accordingly, the study’s specific aims were to: (a) identify risk loci and gene sets/pathways for prevalent ECC and related traits (e.g., caries prevalence and severity) by conducting a multi-ethnic GWAS meta-analysis; (b) determine the prevalence and severity of dental caries, other oral disease outcomes, and putative risk factors for dental caries in a low-income population-based sample of European–, African–, and Latino/Hispanic–American preschool-age children.

## 2. Materials and Methods 

ZOE 2.0 is an observational, cross-sectional, community-based epidemiologic investigation of early childhood oral health with focus on ECC. The study received approval (#14-1992) from the University of North Carolina—Chapel Hill Office of Human Research Ethics IRB on 18 September 2014. The study represents the evolution of previous oral health research initiatives in the public preschool child population in North Carolina, including the ZOE study [11,15] between 2012 and 2013, and the ZOE-pilot study between 2013 and 2014 [16]. Specifically, the ZOE 2.0 study population was children attending public preschools (Head Start) in North Carolina, United States, between August 2016 and February 2019. The federal Head Start program is authorized by the Improving Head Start for School Readiness Act of 2007 with the goal to promote school readiness of children from low-income families. Children were eligible to participate in ZOE 2.0 if they were between the ages of 3–5 years and currently enrolled in a North Carolina Head Start program. Other eligibility criteria were a legal guardian at least 18 years old who was able to understand the study’s informational material and consent documents that were provided in English and Spanish language, and provided their written informed consent. At enrollment, children’s legal guardians completed a one-page, self-administered, paper-and-pen questionnaire that included 15 demographic and oral health-related questions. To enable the production area-level visual summaries and analyses, participants’ residential locations were geocoded using ArcGIS Pro software (Esri, Redlands, CA, USA).

The target analytical sample of ~6000 was set balancing the requirements of adequate power to detect genetic effects and feasibility—it was based upon a goal of >80% power to detect relative effect sizes (i.e., for the binary trait of established ECC case status) of 1.15 for single nucleotide polymorphisms (SNPs) with minor allele frequency (MAF) ≥ 0.45, 1.20 for MAF ≥ 0.20, and 1.30 for MAF ≥ 0.10. We consider these scenarios and plausible detectable effect sizes realistic in light of the only genome-wide significant marker reported for the primary dentition caries to date, rs1594318 (MAF = 0.40, odds ratio = 0.85, *p* = 4.1 × 10^−8^) [12]. 

The study’s design and sampling frame specified procedures for the initial selection of 24 out of the state’s 52 Head Start programs, stratified into four geographical regions of NC. All centers within the invited programs and all children within each center were invited to participate. The selection of programs used probability proportionate to size (PPS) sampling within strata (i.e., 4 geographic regions) and assumed a 75% enrollment rate. Because the enrollment rate after the first year of the study was considerably lower than expected (42%), the study team invited 2–3 additional programs in each region, resulting in the enrollment of 10 additional programs (i.e., a total of 34). Of note, by the end of the study, the cumulative enrollment rate had increased to 62%.

After enrollment, 10 trained and calibrated clinical examiners (registered dental hygienists or dentists) conducted comprehensive examinations of participating children in their preschools. After toothbrushing among all children (and flossing as needed), children’s teeth were examined using portable dental equipment, compressed air, and uniform artificial light and magnification conditions (i.e., custom-fit magnifying loupes with headlights; Orascoptic XV1™ Loupe + Light, Orascoptic, Middleton, WI, USA). Recording of clinical data was done using a Microsoft Access-based (Microsoft Corp., Redmond, WA, USA) custom-written data entry application. A detailed description of the study’s clinical protocol and procedures has been reported by Ginnis et al. [17]. Briefly, tooth surface caries diagnoses were based on modified International Caries Detection and Assessment (ICDAS) criteria [18,19] and made at the levels of health (ICDAS code, 0, representing caries-free tooth surfaces), early stage (ICDAS codes, 1–2, representing initial caries lesions), and established stage (ICDAS codes, 3–6, representing established or severe caries lesions). These stages resemble earlier, historic classifications of caries lesions as “pre-cavitated” (i.e., analogous to early stage) or “cavitated” (i.e., analogous to established/severe) but do not correspond exactly to them—for example, ICDAS 4 lesions may or may not be cavitated, but they are nowadays considered established due to their extension in dentin [19]. Data were summed from all primary tooth surfaces to calculate the dmfs index, a count of primary tooth surfaces that were decayed, missing due to caries or filled/restored (ranging between 0 and 88). Different caries lesion detection thresholds were used to calculate two values of dmfs: early-stage (ICDAS ≥ 1; classic ECC definition [1]) and established (ICDAS ≥ 3) [17]. Classic ECC is the internationally accepted case definition for ECC and includes the earliest detectable lesions compatible with enamel demineralization loss [1,19]. The established definition includes lesions with deeper demineralization and possibly tooth surface loss—as such, it has more clinical and public health relevance and is also the closet to what is measured using the National Health and Nutrition Examination Survey (NHANES) clinical examination protocol [7]. The collection of detailed surface-level data on experience allows for the creation of several other clinical traits for comparisons with previous and future studies, including or excluding restorations (e.g., fillings, crowns) and extracted teeth, as well as clinical patterns of ECC (e.g., pits and fissures, smooth surfaces, proximal surfaces, etc.). Additional clinical examination domains included skeletal and occlusal characteristics, developmental defects of the enamel, dental trauma, and restorative treatment needs. Children’s height and weight were measured using a portable stadiometer (Seca^®^ 213, Seca GmbH & Co. KG, Hamburg, Germany) and a portable digital scale (Doran^®^ DS6150 Remote Indicator Scale, Doran, Batavia, IL, USA) [17]. Age- and sex-specific body mass index (BMI) Z-scores and corresponding BMI categories (i.e., underweight, normal weight, overweight, obese) were calculated using the zanthro program in Stata 16.1 (StataCorp LLC, College Station, TX, United States) and the 2000 Centers for Disease Control (CDC) Growth reference data.

Saliva samples were collected from children using the DNA Genotek Oragene DNA-575 kit (DNA Genotek, Ottawa, Ontario, Canada). This was done for children that presented for a clinical examination. DNA was extracted from these saliva samples, quantitated, and quality assessed using procedures detailed in the study’s genomics analysis protocol [20]. The purified DNA material was carried forward to high-density genotyping at the Center for Inherited Disorders Research (CIDR), at Johns Hopkins University, using the Infinium™ Global Diversity Array (Illumina, San Diego, CA, USA).

Two supragingival plaque samples were also collected for each participant; one pooled sample from all facial/buccal surfaces of the upper right quadrant and one pooled sample from the facial/buccal surfaces of the upper left quadrant. These samples were biobanked for future studies involving metagenomics, metatranscriptomics, and metabolomics [21,22]. To date, ~5% of them (*n* = 300, equally split between ECC cases and non-cases, selected from the first wave of participants and sequenced in 2 batches) were used in pilot, feasibility and discovery studies to facilitate the development of novel multi-omics analysis methods. Detailed, step-by-step descriptions of procedures for collection, transportation, storage, and processing of all biospecimens (i.e., saliva and two plaque samples for each participant) have been made available via three publicly available publications [17,20,21]. 

**Figure 1:**
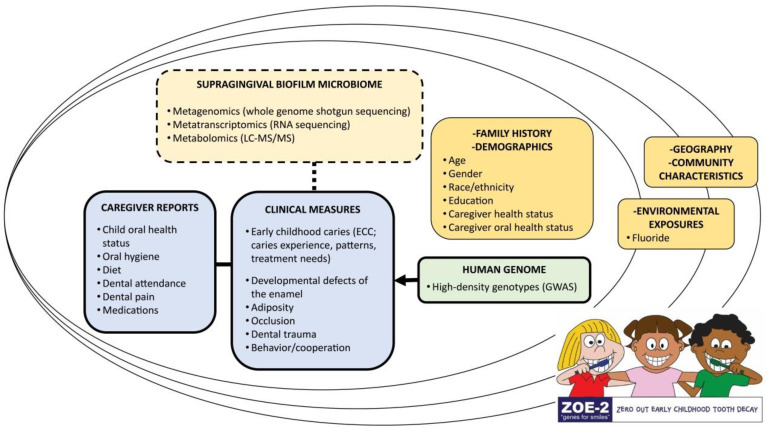
Overview of data domains and measures collected in the ZOE 2.0 study, North Carolina, United States. Adapted with permission from Divaris and Joshi 2020 [20]. To date, supragingival biofilm microbiome analyses have been carried out in a pilot subset of 300 ZOE 2.0 participants (~5% of those with clinical data).

A sample of tap water from the child’s home was obtained after the clinical examination and was used for fluoride ion concentration measurement by the North Carolina State Laboratory of Public Health using the United States Environmental Protection Agency (EPA) 300.0 method. An overview of data domains and measures collected in the study has been previously reported and is illustrated in Figure 1.

Quality assessment and assurance procedures were based upon periodic review of database reports, including but not limited to enrollment data, clinical data, biospecimen manifests, fluoride measurement reports, consents received and signed, physical and scanned questionnaires. Periodic study-wide quality control procedures were implemented both by the study team and an external independent monitor (Rho Inc., Durham, NC, USA), as required by the study sponsor. Targeted reviews of primary records were done by at least two study members in cases where: (a) free-text responses where encoded to study variables (e.g., children’s medication list and measured height and weight); (b) participants’ residential addresses could not be exactly geocoded due to spelling or data entry errors; (c) clinical examiners entered notes in “notes field” to be reviewed by the core study team.

For this cohort profile, descriptive statistics were estimated using Stata 16.1 (StataCorp LLC, College Station, TX, USA) and SAS 9.4 (SAS Institute Inc., Cary, NC, USA). Estimation accounted for the clustered nature of observations (i.e., children’s enrollment in 34 different Head Start programs) via the use of robust Taylor-linearized variances.

## 3. Results

### 3.1. Enrollment of Study Participants

**Figure 2:**
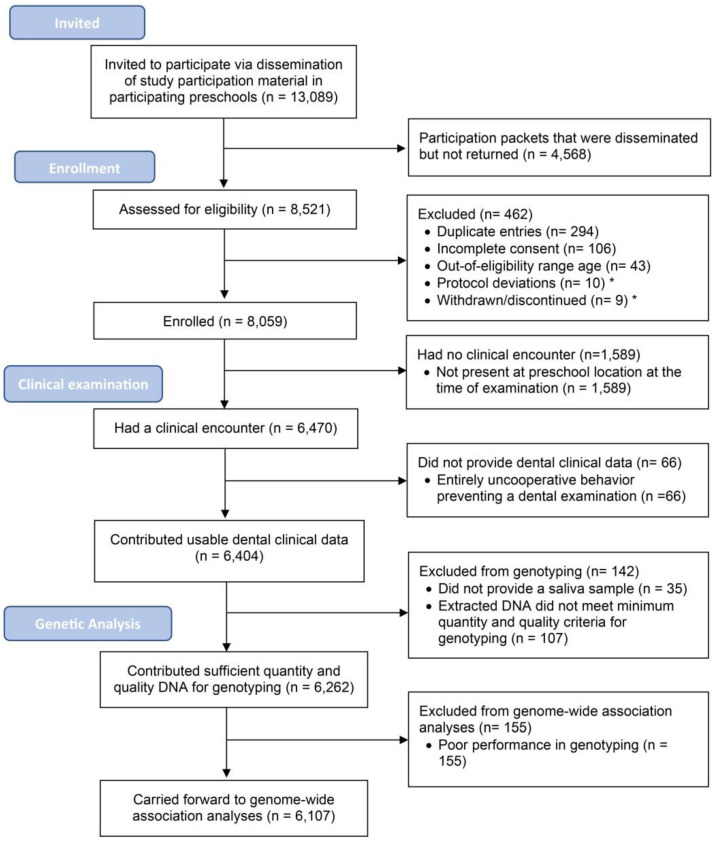
Flowchart of enrollment, clinical examinations, and derivation of the GWAS analytical samples in the ZOE 2.0 study, North Carolina, United States (the 19 participants that are identified with protocol deviations that were withdrawn, or discontinued were initially considered as part of enrollment, but were removed from the study enrollment and do not count towards the reported enrollment figure of 8059).

**Figure 3:**
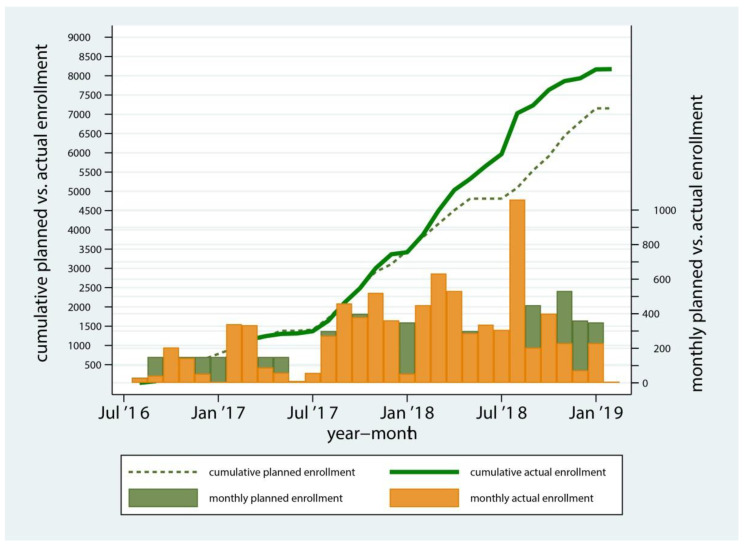
Monthly and cumulative enrollment during the 30-month enrollment period in the ZOE 2.0 study, North Carolina, United States.

The study’s target analytical sample for the conduct of the GWAS was 6000 examined and genotyped children. There were approximately 20,000 children enrolled in the North Carolina public preschool (Head Start) system, the target population. A flowchart describing participants’ enrollment, clinical examination, and the derivation of the GWAS sample is presented in Figure 2. Monthly and cumulative enrollment numbers over time, during the 30-month enrollment period are presented in Figure 3. The study team used information on enrollment and examination rates from pilot studies and from the first year of the study to initially design, and subsequently adjust and optimize the recruitment strategy to meet the target analytical sample size. Approximately 13,000 participants were invited via written material disseminated to Head Start centers and 62% of those (*n* = 8059) were enrolled. The overwhelming reason for exclusion after the assessment of eligibility was duplicate entries (i.e., more than one participation packet for the same participant), followed by incomplete documentation, such as missing or unsigned consent, and being outside the eligible age range.

**Figure 4:**
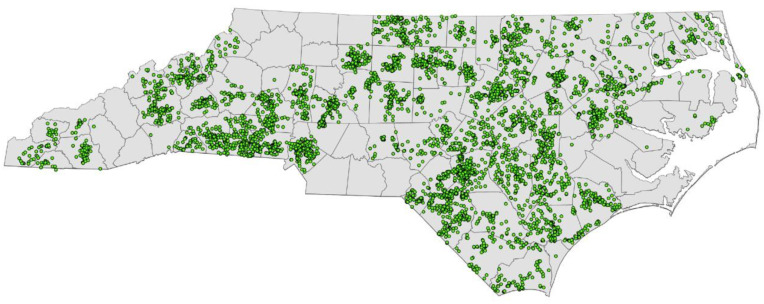
Geographical distribution of participants enrolled in the ZOE 2.0 study across the state of North Carolina, United States.

As illustrated in Figure 4, enrolled participants’ residential locations were spread across North Carolina, and were in 86 (out of a total 100) counties of the state. Enrolled children attended 34 different Head Start programs (clusters), and 260 different Head Start centers nested within these programs. Based on the number and size of programs and centers, the Head Start program was determined as the appropriate level of clustering for analytical purposes.

### 3.2. Clinical Examinations

**Figure 5:**
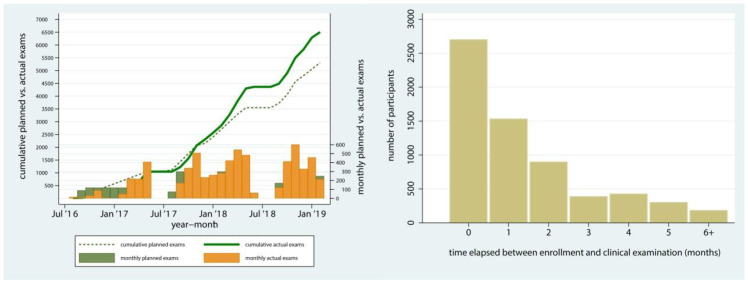
Monthly and cumulative clinical encounters during the 30-month study period and distribution of time elapsed (months) between enrollment and clinical examination in the ZOE 2.0 study, North Carolina, United States.

Eighty percent of enrolled participants (*n* = 6470) were seen for clinical assessments and 99% of those encounters (*n* = 6404) yielded usable clinical information on ECC. Clinical encounters that did not lead to usable clinical data were due to children’s uncooperative behavior, which is not unexpected in this age group. The monthly and cumulative numbers of clinical examinations are presented in Figure 5 (left panel) and generally paralleled enrollment. As illustrated on the right panel of Figure 5, most exams were conducted within 2 months after enrollment; median and mean days elapsed between enrollment and examination were 36 and 57, respectively.

### 3.3. Data Completeness

We considered data completeness by data domain (e.g., questionnaire, clinical examination, biospecimens, water sample return, etc.) and for each individual questionnaire item. As presented in Table 1, data completeness was high for all data domains except for domestic water samples returned. Directly measured concentrations of participants’ domestic water source fluoride content are available for 24% of those with ECC data. Fluoride measurement was done for virtually all water samples received—the most common reason for missing fluoride measurements of water samples received was over 30 days elapsed between the reported water sample collection and receipt of the sample in the lab. A smaller proportion of samples were not carried forward to measurement due to issues with labeling or shipping (e.g., leaked during transportation). 

Nearly all examined participants (98–99%) provided saliva and supragingival plaque samples. Human genomic DNA of adequate quality and quantity was available for 98% of those who provided saliva samples. A similarly high percentage of samples with adequate quantity and quality DNA (98%; *n* = 6107) performed satisfactorily during genotyping and are eligible to be carried forward to a GWAS.

Similarly, data missingness was rare when considering responses to individual questionnaire items, with most items having 0 to 2% missing data. As presented in Table 2, the highest proportion of missing responses was noted for the question “What is your child’s race” which was commonly left incomplete among Spanish language respondents and participants of Hispanic ethnicity. Of note, 11% of participants (*n* = 923) elected to complete study materials in the Spanish language. In terms of respondent-level missingness, 88% of respondents had no missing questionnaire responses; 9.4% had one, 1.4% had two, and 1.2% had three or more. Missingness was higher among those who completed the Spanish language questionnaire: 26% had at least one missing response, and 7% had at least two missing responses compared to 10% with at least one missing response and 2% with at least two missing responses among those who completed the English language questionnaire.

### 3.4. Protocol Deviations

A total of 10 protocol deviations occurred during the course of the study. None of them affected participant safety or the interpretability of the study data. Nine of these protocol deviations were due to children who were marginally outside the age range for eligibility and were inadvertently clinically examined during the first year of the study. The tenth protocol deviation pertained to the clinical examination of an incompletely consented participant. These participants were subsequently rendered ineligible and were reported as protocol deviations to the overseeing UNC-Chapel Hill IRB who determined that they did not increase participants’ risk. These protocol deviations were also reported to the sponsor (National Institutes of Health). It was determined that these deviations did not meet the criteria for Unanticipated Problems Involving Risks to Subjects or Others (UPIRSO), or other noncompliance, and the study team’s corrective action plan in response to these protocol deviations was deemed acceptable.

### 3.5. Enrolled and Examined Participants’ Demographic Characteristics 

Enrolled participants were mostly 4-year-olds (i.e., their mean and median age at enrollment was 52 months). African–Americans were the most represented racial group (48%), followed by whites (22%), and those of more than one race. One fifth (*n* = 1611) of respondents were of Hispanic ethnicity. Importantly, as presented in Table 3, there were no differences in the demographic profile of participants enrolled and those who were clinically examined and provided information for ECC. 

### 3.6. Main Study Outcomes—ECC

ECC was common in this community-based sample of preschool-age children. As presented in Table 4, more than half of examined children (54%, *n* = 3465) were ECC cases at the established/severe caries lesion detection threshold (ICDAS ≥ 3).

The dmfs index was zero-inflated and right-skewed, with a sample mean of 8 and a standard deviation of 14. Because a child’s dmfs index cannot reduce within this age range while the disease may progress, caries prevalence was substantially higher among older children (e.g., 45% among 3-year-olds vs. 61% among 5-year-olds). Children in the least represented racial groups and those of more than one race had higher ECC prevalence and burden compared to African–Americans and whites. Similarly, those of Hispanic ethnicity had higher ECC prevalence and burden compared to their non-Hispanic counterparts. These patterns were similar but less pronounced when considering the early-stage definition of ECC, defined at the ICDAS ≥ 1 caries lesion detection threshold. Ninety-two percent of children were ECC cases according to this definition, with a mean dmfs of 15. Differences in ECC prevalence between age groups were less pronounced using this definition.

In terms of BMI related outcomes, 10% of children (*n* = 624) were found to be underweight (i.e., <5th BMI percentile for age and sex), 68% had normal weight, 13% were overweight, and 9% (*n* = 588) were obese (i.e., ≥95th BMI percentile for age and sex). 

## 4. Discussion

The successful completion of the data collection for the ZOE 2.0 GWAS holds promise for new insights about genomic influences on ECC. We anticipate that this cross-sectional study’s findings will offer novel molecular, clinical, and contextual insights for prevalent ECC.

Looking ahead, ample opportunities now exist for the validation of biomarkers (e.g., microbiome and genome) using prospective follow-up studies that are envisaged for this cohort [20]. The study enrolled a sizable sample of over 8000 preschool-age children for which guardian-provided measures of child oral health were available and approximately 6500 children were clinically examined. Virtually all examined children provided saliva and supragingival plaque (i.e., biofilm) samples enabling a GWAS of ECC, microbiome, and other multi-omics studies.

We found that 54% of children examined in this low-income, community-based sample had caries experience at the established ECC threshold and children had, on average, eight caries-affected tooth surfaces per child. The observed ECC prevalence is greater than national estimates. According to NHANES 2011–2016 data [7], 23% of 2–5-year-old children in the United States had caries experience at the cavitation threshold, and prevalence was 34% among children living below the federal poverty level. Our finding of higher ECC prevalence among Hispanic (61%) vs. non-Hispanic (52%) participants mirrors the nationally-representative data, wherein Mexican–Americans had higher ECC prevalence compared to non-Hispanic whites and African–Americans (33% versus 18% and 28%, respectively). However, it is noteworthy that non-Hispanic white and African–American children had identical ECC prevalence in our study.

A key feature of ZOE 2.0 is its community-based design and the inclusion of substantial proportions of children from minority groups—this is important from a public health standpoint, because estimates of ECC experience can be reasonably generalized to this demographic group of public preschool-attending children in North Carolina. This feature is also important for the planned oral health genomics studies, where the under-representation of racial/ethnic minorities is an important problem [23]. We plan to account for population stratification (i.e., ancestry) by computing 10–20 ancestry principal components using Genome-wide Complex Trait Analysis (GCTA) software [24]. We will carry out genome-wide association analyses using Genetic Association Analysis Under Complex Survey Sampling (SUGEN) software [25], implementing established [26,27] and recently developed strategies for multi-ethnic GWAS [28,29].

The study also benefits from its collection of detailed tooth surface-level clinical data, enabling both “deep and wide” types of analyses [30]—these may include interrogations of the consensus ECC case definitions [1] and harmonization with consortia [12] and databases with similar phenotypes and genetic data. At the same time, the already-collected, detailed clinical data and the stored biospecimens can be used to generate multi-omics information (i.e., human genome, metagenome, transcriptome, and metabolome) and thus enable the creation and study of more refined, data-driven or biologically informed disease subtypes. We have previously demonstrated that biologically informed or biologically enriched dental traits (e.g., including information from the oral microbiome) contain more heritable (i.e., genetically explained) variance, and as such may be more promising GWAS traits [31]. While this hypothesis has not been systematically tested for dental caries and ECC, we posit that information on microbial taxonomy, activity, and metabolism that can be obtained from the study’s supragingival plaque samples will enhance our ability to understand biological pathways contributing to ECC and its postulated subtypes [6,32,33]. It must also be stressed that genetic risk factors do not operate in a vacuum and interactions with important behavioral and environmental factors (e.g., consumption of sugar-containing snacks and beverages and exposure to optimally fluoridated water) are likely to be informative for the study of ECC. To facilitate data sharing, clinical, genomics, and microbiome data generated in ZOE 2.0 are being deposited in the dbGaP repository [34], under the umbrella study name Trans-Omics for Precision Dentistry and Early Childhood Caries or TOPDECC (accession: phs002232.v1.p1).

The low rate of families’ rerunning home water samples was a challenge that was identified during the first year of the study. Two mitigating strategies exist to address this missing data problem. First, the parent questionnaire includes a question about the primary source of home water for the child (e.g., tap, well, bottled water, etc.); this information is important because most well water in NC and most bottled water is non-fluoridated, whereas most community water in NC is fluoridated. Second, a geographic information systems (GIS)-based imputation method is being developed for the purpose of inferring other participants’ fluoride levels based on their residential address and the observed data.

Childhood oral disease has important, multi-level consequences for children, their families, the communities they live in, and the health systems that serve them [35]. ECC is both a person-level clinical problem and an important public health issue characterized by pronounced, persistent disparities [36]. It is thus important that efforts and resources are invested in both precision health-driven programs [37,38] that aim to study and operationalize individual susceptibility, as well as precision public health initiatives [22,39], aiming to inform or develop community interventions and policy. ZOE 2.0 aims to foster the generation of novel molecular, clinical, behavioral, and contextual insights into early childhood oral health and ECC. It is envisaged that this new information will serve as the basis of advancing precision oral health and care, and precision dental public health. Future longitudinal studies, prospectively evaluating ZOE 2.0 cohort participants have the potential to add to the knowledge base of dental caries incidence and progression, validate genetic, microbial, or metabolomic biomarkers collected at baseline, and provide valuable information on prospectively-assessed oral health outcomes in childhood and adolescence. Taken together, this information can facilitate the refinement of existing disease taxonomies and the improvement of current caries risk assessment tools [6,40].

Importantly, the ZOE 2.0 study is a community-based epidemiologic study of early childhood oral health that enables the interrogation of both upstream influences (e.g., geography, social, and area-level determinants) and proximal determinants (e.g., oral health-related behaviors, biomarkers, etc.). In fact, the availability of detailed social and biological information on several thousands of participants with comprehensive dental examination data, presents opportunities for the examination of the crosstalk or links between these different levels [41]. Aside from addressing important scientific questions, we envision that information generated from the study can also inform ECC surveillance, public health planning, and resource allocation. It may also serve as the basis for the development of preventive or therapeutic intervention studies, and ultimately influence actions at the policy level. We envision that the publication of the study’s clinical, genomics, and microbiome protocols, as well as the public sharing of its human genomics and biofilm multi-omics data will facilitate the conduct of similar studies in other areas of the world.

## 5. Conclusions

The ZOE 2.0 cohort is a sizable, community-based, multi-ethnic, genetic epidemiologic study of early childhood oral health. The study’s publicly available, de-identified clinical, behavioral, human genomics, and biofilm multi-omics data create a rich information source that can help answer important questions about the multi-level determinants of early childhood oral health.

## Figures and Tables

**Table 1:** Numbers and percentages of participants with non-missing data by domain.

	*n* (%)
Questionnaire data (among those with verified, consented enrollment)	8054/8059 99.9%
Usable (i.e., non-missing) clinical dental data (among those with attempted clinical exams)	6404/6470 99%
Usable (i.e., non-missing) anthropometric (i.e., height/weight) data (among those with attempted clinical exams)	6442/6470 99%
Saliva collected (among those that had exams and have non-missing clinical data)	6369/6404 99%
Supragingival plaque collected (among those that had exams and have non-missing clinical data)	6271/6404 98%
Adequate quality and quantity of human DNA available (among those with non-missing clinical data that provided saliva samples)	6262/6369 98%
Acceptable performance during genotyping (among those with adequate quality and quantity of human DNA)	6107/6262 98%
Water sample provided for fluoride measurement (among those with attempted clinical exams)	1613/6470 25%
Usable fluoride concentration information (among those who provided a domestic water sample)	1518/1613 94%
Fluoride concentration data available (among those with clinical data)	1518/6404 24%
Fluoride concentration and geocoding data available (among those with clinical data)	1501/6404 23%

**Table 2:** Percentage of missing responses in individual questionnaire items.

Question	%
Who brushes your child’s teeth at home?	0.4
How often are your child’s teeth brushed?	0.4
Is toothpaste with fluoride used every time your child’s teeth are brushed?	1.1
How many snacks and drinks containing sugar does your child usually have between meals?	0.5
Has your child ever gone to the dentist?	1.1
Does your child usually see a dentist for a check-up or because of dental problems?	1.3
Has your child ever been put to bed with a bottle containing something other than water?	2.1
How would you describe the condition of your child’s mouth and teeth?	1.2
How would you describe the condition of your mouth and teeth?	2.6
Has your child ever had a toothache or other dental pain (not from teething)?	1.2
Has your child been given an over-the-counter medication during the last 30 days?	1.2
What is the highest grade-level of schooling you have completed?	2.6
Are you of Hispanic or Latino origin?	1.1
What is your child’s race?	2.9
What is the primary source of your home drinking water for your child?	1.1

**Table 3:** Demographic characteristics of all participants enrolled and all participants that provided early childhood caries (ECC) information via clinical examinations in the ZOE 2.0 study, North Carolina, 2016–2019.

	All Enrolled	w/ECC Information	*p* *
	*n* (Column %)	*n* (Column %)	
Entire Sample	8059 (100)	6404 (100)	
Sex ^†^			
male	4000 (50)	3189 (50)	0.6
female	4057 (50)	3215 (50)	
missing	2	0	
Age at enrollment (years)			0.2
3	2568 (32)	1992 (31)	
4	4234 (53)	3375 (53)	
5	1257 (16)	1037 (16)	
(months), mean (SD)	52 (7.5)	52 (7.4)	0.2
Race			
African–American	3872 (48)	3094 (48)	0.7
American–Indian or Alaskan Native	236 (3)	186 (3)	
Asian	42 (1)	32 (1)	
Native Hawaiian or other Pacific Islander	7 (0.1)	4 (0.1)	
White	1765 (22)	1385 (22)	
>1 race	1067 (13)	835 (13)	
other/missing	1070 (13)	868 (13)	
Hispanic ethnicity			0.8
yes	1611 (20)	1291 (20)	
no	6355 (80)	5042 (80)	
missing	93	71	

ECC, Early Childhood Caries; SD, Standard Deviation. * Bivariate tests accounting for the clustered nature of the data. †The parent questionnaire asked whether the participating child was a boy or girl; subsequently, biological sex was determined via genotyping.

**Table 4:** Estimates of early childhood caries (ECC) experience (case status, defined as one or more decayed, missing, or filled tooth surface (dmfs) due to dental caries) and burden (dmfs) overall and according to participants’ demographic characteristics in the ZOE 2.0 study, North Carolina, 2016–2019. Two thresholds of caries lesion detection according to the International Caries Detection and Assessment System (ICDAS) are used—ICDAS ≥ 3 (established ECC) and ICDAS ≥ 1 (classic ECC).

	Established ECC, Defined at the ICDAS ≥ 3 threshold		Classic ECC, Defined at the ICDAS ≥ 1 threshold	
	dmfs > 0, *n* (row %)	dmfs, mean (se*)	dmfs>0, *n* (row %)	dmfs, mean (se*)
Entire Sample	3465 (54)	8 (0.6)	5882 (92)	15 (0.7)
Sex				
male	1748 (55)	8 (0.5)	2935 (92)	16 (0.6)
female	1717 (53)	8 (0.7)	2947 (92)	15 (0.7)
Age (years)				
3	673 (45)	5 (0.4)	1347 (90)	13 (0.6)
4	1836 (55)	8 (0.6)	3084 (92)	15 (0.7)
5	956 (61)	11 (0.8)	1451 (93)	17 (0.9)
Race				
African–American	1622 (52)	7 (0.5)	2845 (92)	14 (0.6)
American–Indian or Alaskan Native	127 (68)	15 (1.6)	180 (97)	22 (1.3)
Asian	24 (75)	16 (3.3)	32 (100)	23 (3.5)
Native Hawaiian or other Pacific Islander	4 (100)	21 (6.7)	4 (100)	30 (8.0)
White	725 (52)	8 (0.6)	1251 (90)	15 (0.9)
>1 race	426 (51)	7 (0.6)	741 (89)	13 (0.7)
other	534 (62)	11 (1.0)	825 (95)	18 (1.1)
Hispanic ethnicity				
yes	788 (61)	11 (1.0)	1209 (94)	18 (1.2)
no	2643 (52)	7 (0.5)	4604 (91)	14 (0.6)
missing	34 (48)	8 (2.4)	69 (97)	16 (2.4)

ECC, Early Childhood Caries; ICDAS, International Caries Detection and Assessment System; dmfs, decayed, missing, filled/restored tooth surfaces due to caries. *se—linearized standard errors accounting for the clustered nature of the data (i.e., attendance in 34 different public preschool programs).
